# Deep Brain Stimulation of the Pedunculopontine Tegmental Nucleus (PPN) Influences Visual Contrast Sensitivity in Human Observers

**DOI:** 10.1371/journal.pone.0155206

**Published:** 2016-05-11

**Authors:** Hendrik Strumpf, Toemme Noesselt, Mircea Ariel Schoenfeld, Jürgen Voges, Patricia Panther, Joern Kaufmann, Hans-Jochen Heinze, Jens-Max Hopf

**Affiliations:** 1 Leibniz Institute for Neurobiology, Magdeburg, Germany; 2 Institute for Biological Psychology, Otto-von-Guericke University, Magdeburg, Germany; 3 Clinic for Neurology, Otto-von-Guericke University, Magdeburg, Germany; 4 Clinic for Stereotactic Neurosurgery, Otto-von-Guericke University, Magdeburg, Germany; 5 Kliniken Schmieder, Allensbach, Germany; University of Bologna, ITALY

## Abstract

The parapontine nucleus of the thalamus (PPN) is a neuromodulatory midbrain structure with widespread connectivity to cortical and subcortical motor structures, as well as the spinal cord. The PPN also projects to the thalamus, including visual relay nuclei like the LGN and the pulvinar. Moreover, there is intense connectivity with sensory structures of the tegmentum in particular with the superior colliculus (SC). Given the existence and abundance of projections to visual sensory structures, it is likely that activity in the PPN has some modulatory influence on visual sensory selection. Here we address this possibility by measuring the visual discrimination performance (luminance contrast thresholds) in a group of patients with Parkinson’s Disease (PD) treated with deep-brain stimulation (DBS) of the PPN to control gait and postural motor deficits. In each patient we measured the luminance-contrast threshold of being able to discriminate an orientation-target (Gabor-grating) as a function of stimulation frequency (high 60Hz, low 8/10, no stimulation). Thresholds were determined using a standard staircase-protocol that is based on parameter estimation by sequential testing (PEST). We observed that under low frequency stimulation thresholds increased relative to no and high frequency stimulation in five out of six patients, suggesting that DBS of the PPN has a frequency-dependent impact on visual selection processes at a rather elementary perceptual level.

## Introduction

The pedunculopontine tegmental nucleus (PPN) is an important component of the general reticular activation system (RAS) involved in the regulation of wakefulness, REM sleep, locomotion, as well as a variety of other cognitive functions (for overviews see [1–4]. Its wide range of modulatory influences is paralleled by a distributed connectivity to many subcortical and cortical areas in mammals [5, 6] including the human [7, 8]. The PPN shows strong connectivity with the basal ganglia (globus pallidus internus GPi) [2], the subthalamic nucleus (STN), the substantia nigra (SN) [5, 6, 9], as well as with the spinal chord. Stimulation of the PPN revealed an influence on locomotion and postural control in the monkey [10–12] with reversible inactivation and lesions producing Parkinson-like symptoms [13, 14]. These and clinical observations in patients suggested that the PPN may be a relevant brain structure involved in producing symptoms of Parkinson’s disease (PD) in humans [15–18]. The PPN was accordingly selected as a target site for deep brain stimulation with the goal to control PD associated gait and postural disturbances [15, 16, 19–23].

Beyond its role in locomotion, the PPN has been shown to influence oculomotor performance and attention in rodents and monkeys [24–27]. In the latter, PPN neurons displaying saccade- and fixation-related firing patterns could be documented [26]. Moreover, the PPN is known to project to all thalamic nuclei including sensory relay nuclei [28]. Hence, there is a potential role for the PPN in modulating attention and perceptual processes more directly via cholinergic connectivity with relay nuclei of the thalamus as the lateral and medial geniculate nucleus (LGN, MGN) or the pulvinar [29]. The majority of cholinergic brain-stem projections to the LGN in fact come from the PPN [30] and it has been hypothesized that respective projections contribute to the modulation of thalamo-cortical transmission (thalamic gating) underlying selective attention [28]. One such gating pathway could rely on projections to the thalamic reticular nucleus (TRN). The TRN, a structure closely linked to the ARAS, has been suggested to act as a ‘guardian of the gateway to the cortex’ [31]. The ‘guardian’ role of the TRN was recently confirmed by direct experimental evidence in the monkey [32, 33]. Another pathway could involve the PPN’s cholinergic connectivity with the superior colliculus (SC) [25, 34]. The pivotal role of the SC in visual attention has been well documented in animals [35–37]. In particular, it was shown that subthreshold microstimulation of the SC can increase the monkey’s contrast sensitivity of discriminating motion direction [38]–a stimulation effect mimicking the operation of selective visual attention. Furthermore, the SC has been implied to mediate perceptual and emotional sensitivity in non-conscious vision like in blindsight [39, 40]. Finally, PPN effects on perceptual sensitivity and attention may arise from its connectivity with the SN, where dopaminergic projections to the frontal lobe (in particular the frontal eye field, FEF) and/or other structures (e.g. the SC) modulate attentional selectivity in extrastriate visual cortex areas. For example, subthreshold stimulation of FEF neurons was shown to modulate the firing response of extrastriate neurons (V4) whose receptive field (RF) corresponded with the RF of the stimulated FEF neurons [41, 42]. Moreover, microiontophorectic administration of a D1-receptor antagonist in the FEF increased the magnitude and selectivity (to orientation) of responses in corresponding V4 neurons [43].

**Fig 1:**
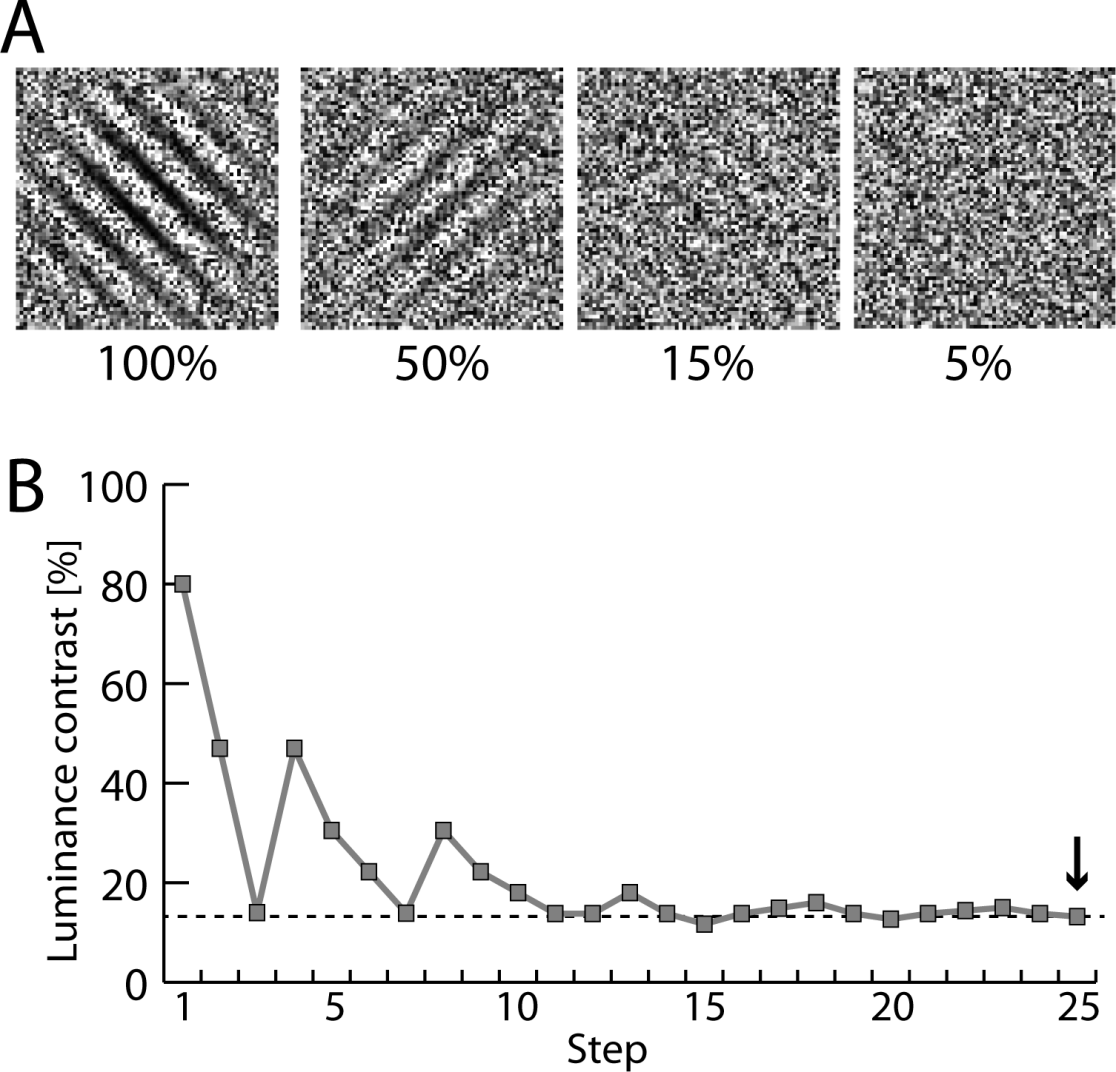
Example stimuli and staircase measurement. (A) Example stimuli showing oriented Gabor-gratings embedded in pixel noise at decreasing luminance contrast (100%, 50%, 15%, 5%). (B) Example of one staircase measurement in patient P1. The staircase protocol was based on a modified version of the PEST (Parameter Estimation by Sequential Testing) procedure. The last contrast-value (arrow) was taken as the measure of the contrast-threshold of a given staircase run.

In summary, several lines of experimental evidence hint at the possibility that neuromodulatory projections from the PPN have specific influence on attention and visual sensory selection. This possibility, however, has not been experimentally investigated yet. Here we address this possibility in patients suffering from Parkinson’s disease, which underwent deep-brain stimulation (DBS) of the PPN to alleviate severe gait/postural disturbances. Specifically, we developed an experimental test design that allowed us to determine the discrimination threshold of a noise-embedded visual stimulus under different DBS frequencies. Subjects were to report the orientation of a Gabor grating embedded in random pixel noise (Fig 1A). The luminance-contrast of the Gabor varied from trial to trial following a staircase protocol (PEST procedure, see Methods). Below a certain luminance contrast, the grating ‘vanished’ into the noise with the subjects’ discrimination performance approaching chance level. The staircase was constructed to converge at the threshold contrast at which subjects were just able to perform the orientation discrimination correctly. We asked whether microstimulation of the PPN would affect detection thresholds, and if yes, how the effect on thresholds would vary with stimulation frequency.

## Materials and Methods

### Participants

Eight patients (5 female, 3 male) suffering from PD participated in the study. Two subjects could not be included in the final data analysis due to insufficient data collection (performance breakup after a few runs). All patients were inpatients of the Department of Stereotactic Surgery of the University Clinic for Neurology, Magdeburg. All participating patients gave written consent, volunteered and were free to stop the experiment at any time. None of the patients had a history of psychiatric or neurological diseases other than PD. No patient showed signs of depression or dementia at the time of experimentation (all MDRS scores > 131). All patient were medicated with L-Dopa and were tested under on-going medication. Three patients received additional bilateral DBS (Table 1) of the subthalamic nucleus (STN), which was always on during testing. Ethics Statement: The experiment was approved by the Ethics Council of the Medical Department of the University of Magdeburg.

**Table 1:** Summary of stimulation schedule and parameters of each patient included in the data analysis.

Pat.	Age	Stim.			∂t		Dur.	Int.	Site	AaS	Co-DBS
		1.	2.	3.	1.-2.	2.-3.					
P1	50	60	10	no	3	2	60	1.5	l/r	3	STN(l/r)
P3	56	no	10	60	4	2	60	2.0	r	4	STN(l/r)
P4	71	20	no	/	0	/	60	2.0	l/r	6	
P6	67	60	no	8	1	1	60	2.5	l/r	13	
P7	73	8	60	no	2	2	60	1.0	l/r	13	
P8	68	8	no	60	1	2	60	2.0	l/r	9	STN(l/r)

Pat., patient; Stim., frequency of stimulation (Hz) at the first (1.) second (2.) and third (3.) test session; ∂t, time (days) between the first and second (1.-2.) and the second and third session (2.-3.); Dur., duration of stimulation (μs); Int., intensity of stimulation (V); Site, electrode placement in right (r) or both (l/r) hemispheres; AaS, assessment after surgery, the time (months) testing was performed after placing the electrodes; STN, subthalamic nucleus

**Fig 2:**
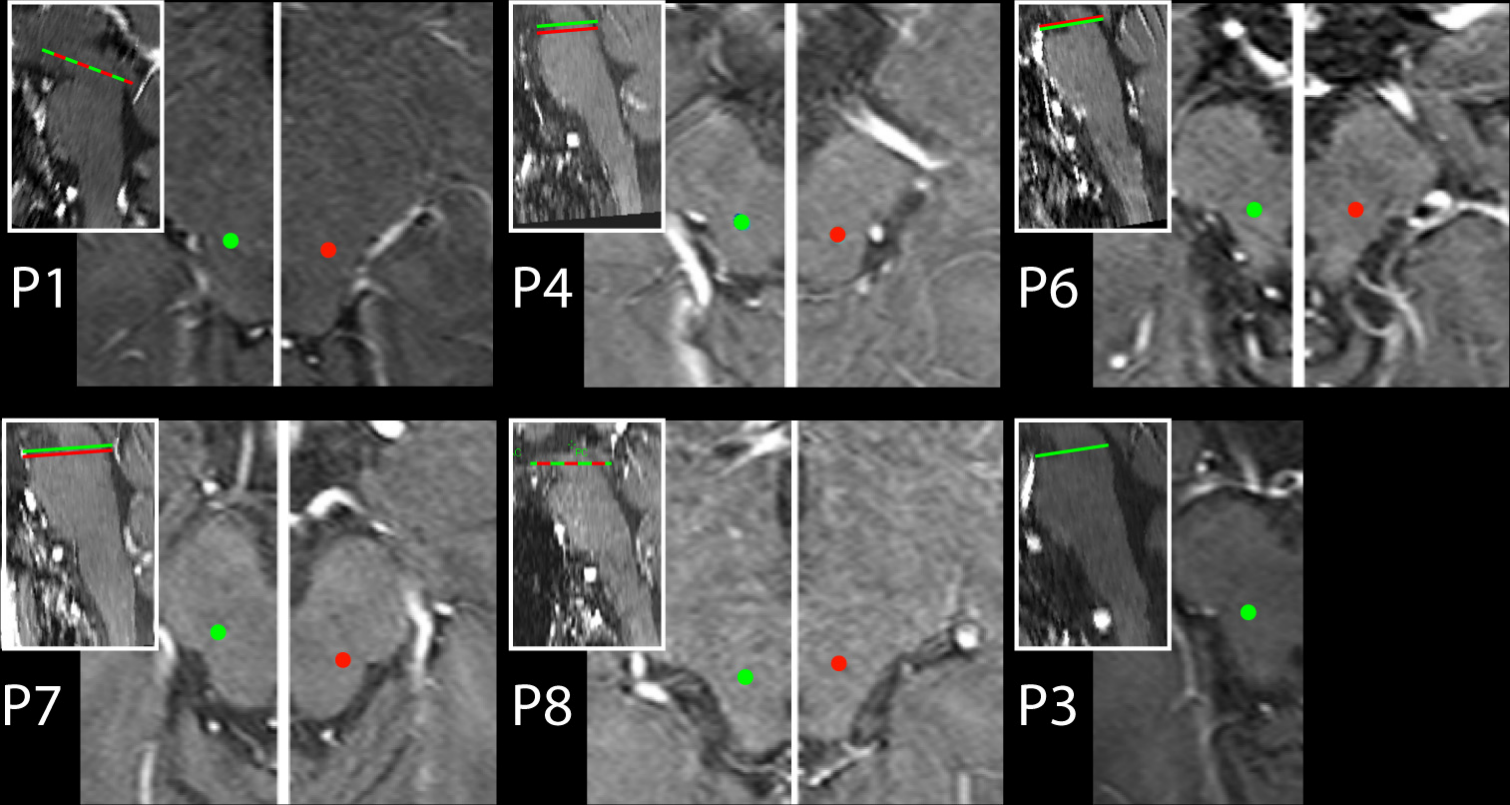
DBS electrode contact locations. The location of the active electrode contacts in the right (green dots) and left (red dots) PPN region. Shown are axial MRI transsections in each patient. Left and right side transsections display different planes along the rostro-caudal axis in P4, P6, and P7. Respective planes are highlighted by green (right side) and red lines (left side) in the inserts showing midline sagittal transsections.

DBS-electrodes (Metronic Model 3389, 4 platinum–iridium cylindrical contacts, diameter/length 1.27/1.5 mm each, edge-to-edge separation 0.5 mm) were placed bilaterally in all but one patient (P3), where only a right-side electrode was implanted. Electrode placement followed standard stereotactic techniques [44, 45] which involved MRI-guided stereotaxy together with microelectrode recordings to aid localization physiologically. The position of macroelectrodes was determined using coordinates of the Atlas for Stereotaxy of the Human Brain [46] which were then individually adjusted through visualization of the patient’s PPN region in a Proton-density weighted MR image [47]. Fig 2 summarizes the position of active electrode contacts in axial slices of each patient. The ultimate stimulation contacts and parameters were individually defined (Table 1) to gain best results in alleviating motor/postural performance deficits.

### DBS protocol

Table 1 summarizes the time of surgery prior to behavioural testing as well as the stimulation protocol and parameters for each subject. Five patients (P1, P3, P6, P7, and P8) were tested under low-frequency stimulation (**LFS**, 8 or 10 Hz), high-frequency stimulation (**HFS**, 60 Hz), or no stimulation (**NoS**) on different days. For clinical reasons, one patient (P4) was not available for testing under 10Hz conditions. Instead, we could obtain performance data under 20 Hz LFS and under NoS conditions. Note, the stimulation protocol and parameters as well as their changes followed therapeutic considerations aiming at improving gait disturbances in the patients. Parameter settings, their variation and randomization as well as the time of testing were not under direct control by the experimenters.

### Stimuli and Procedure

Examples of stimulus frames containing Gabor gratings with different luminance contrast are shown in Fig 1A. The Gabor patch was always embedded in independent pixel noise (random distribution of pixels with grey values from black to white), with each patch containing 250 x 250 pixels (on screen extension: 6.6° x 6.6° of visual angle). The orientation of the Gabor grating was either tilted 45° clockwise or counter-clockwise from vertical, with subjects being required to report the orientation with a two-alternative button press of the right hand (left vs. right: index vs. middle finger). On twenty percent of the trials the target was absent and subjects had to press a third button. Target-absent trials were included to control for the possibility that subjects performed in a random manner when approaching lower contrast levels. Accuracy but not response time was stressed. The duration of stimulus frame presentation was varied from patient to patient (ranging from 1200–2500 ms) to guarantee that the task could be performed properly. Subjects responded in a self-paced trial-by-trial manner, with subsequent frames appearing after a randomized ISI of 800–1300 ms (rectangular distribution) after the manual response. On each trial the luminance contrast of the Gabor was varied according to a staircase-protocol (PEST, Parameter Estimation by Sequential Testing), allowing us to determine the individual contrast level (discrimination threshold) at which the subject could just detect and discriminate the Gabor reliably. The PEST protocol [48] was slightly modified in that each staircase started at the 80% contrast level with the first downward step being a 33% reduction of contrast instead of a 50% reduction. Fig 1B shows one example of a staircase performed by patient P1. To obtain reliable estimates, each subject performed at least 14 staircases per stimulation condition. The contrast level at which the given staircase settled was taken as estimate of the discrimination threshold. As summarized in Table 1 the stimulation conditions (NoS, LFS, HFS) were run on separate days, with the order of stimulation condition changing between subjects.

### Statistical data validation

Statistical validation of the grand average responses over subjects was performed using the Friedman test with the 3-level factor stimulation (HFS,LFS,NoS). Subsequent post-hoc pairwise comparisons were performed using Wilcoxon signed-rank tests. The subjects’ individual response patterns were statistically analysed using one-way repeated measures ANOVAs with the 3-level factor stimulation condition (HFS,LFS,NoS) and individual staircases serving as samples. Violations of data sphericity were corrected with the Greenhouse-Geisser epsilon. Corrected alpha-levels are reported.

## Results

**Fig 3:**
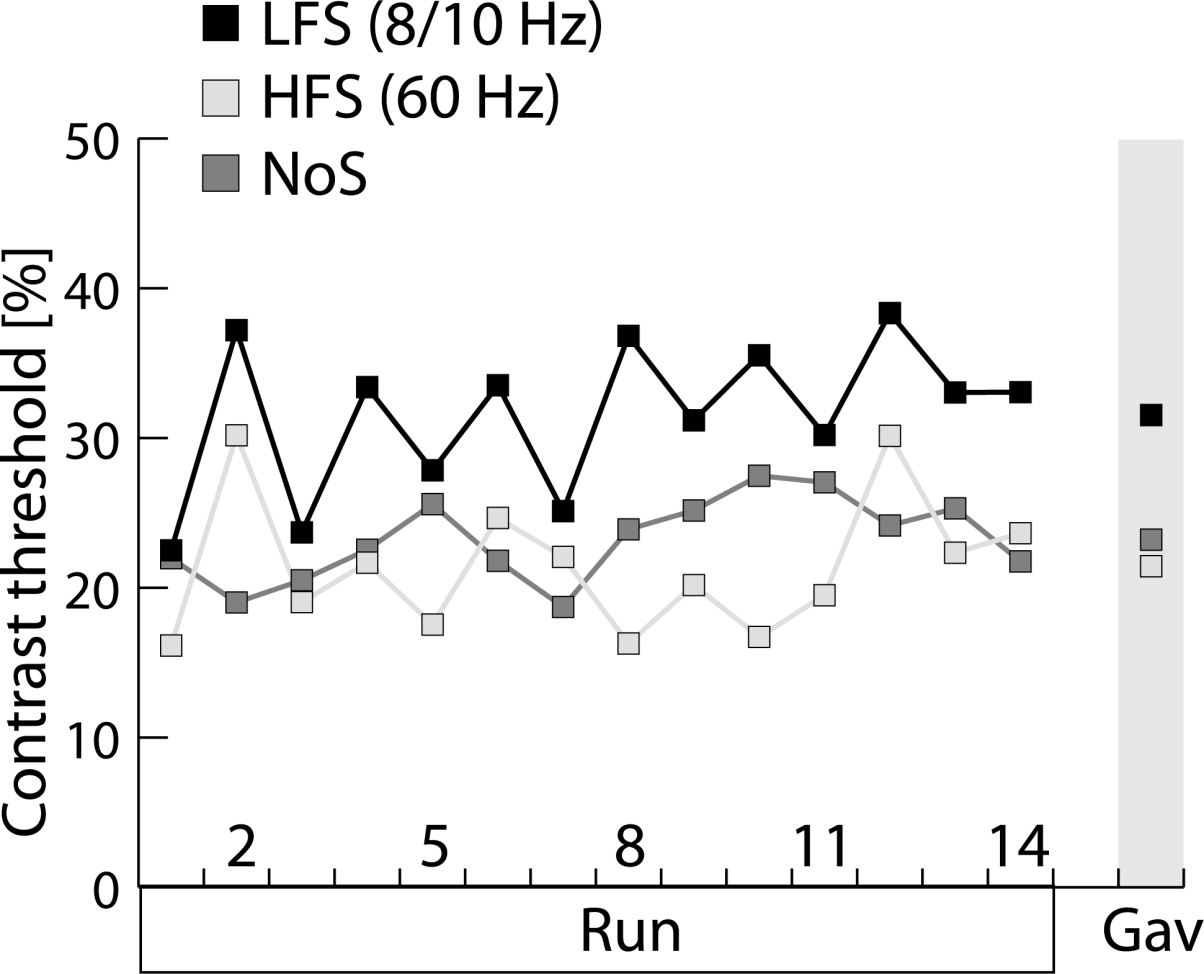
Contrast-threshold measures. Average contrast-threshold measures (over patients, n = 5) on fourteen successive staircase runs under low-frequency (LFS, black), high-frequency (HFS, bright grey) and no stimulation (NoS, dark grey). Grand average (Gav) threshold measures over all subjects and staircases are shown on the right side.

Fig 3 shows average (n = 5 subjects, P4 not included) contrast thresholds of the LFS (black), the HFS (bright grey), and the NoS (dark grey) condition for subsequent staircases (n = 14) of the experimental sessions. Note, subject P1 performed 20 staircases, for consistency only the first 14 staircases entered into the average. The most obvious effect of stimulation is an overall threshold increase for the LFS relative to the NoS and HFS conditions which is consistently present throughout the experiment. That is, LFS shows an average contrast-threshold level (31.5%) that is roughly 10% higher than for the NoS (23%) and HFS condition (19.7%). For statistical data validation a Friedman test with the 3-level factor Stimulation (LFS, HFS, NoS) was computed, which yielded a significant effect (p = 0.041). Post-hoc pairwise comparisons (Wilcoxon signed-rank test) revealed that there was a significant difference between LFS and HFS (p = 0.043) and a trend towards a difference between LFS and NoS (p = 0.08). HFS and NoS, however, were not statistically different (p = 0.345).

Considering the performance in individual patients, the effect of stimulation condition (HFS,LFS,NoS) was significant (p<0.05) in all (P1, P4, P6, P7, P8) but one patient (P3) in which the effect did not reach significance (p = 0.102). The performance decrement for the LFS relative to the NoS condition was present in four patients (P1, P3, P6, P8) tested with 8/10 Hz and 60 Hz stimulation. In the one patient (P4) tested under 20 Hz and NoS conditions, a clear performance decrement was seen for the 20 Hz stimulation (arguably a low frequency condition) relative to the NoS condition. Only P7 showed a performance pattern where LFS was associated with reduced and HFS with increased contrast thresholds relative to NoS, respectively. As visible in Fig 1 the electrode placement in P7 and P4 was slightly more caudal relative to the other patients. The stimulation parameters in P7 were, however, not special. We can therefore only speculate (see Discussion) about what accounts for the ‘reverse’ response pattern in this patient.

## Discussion

In this study we aimed at investigating whether DBS of the PPN influences visual discrimination performance as indexed by the threshold of detecting a Gabor grating embedded in visual noise. We reasoned that PPN stimulation may influence discrimination performance, because it is part of the RAS with widespread cholinergic connections to many subcortical and cortical structures directly or indirectly involved in visual attention [2, 5, 6, 9]. We indeed observed that DBS influenced discrimination performance in form of a decrement (elevated contrast thresholds) under LFS in four patients. In one patient such decrement was also present, but did not reach significance. One patient showed a performance increment under LFS.

The fact that discrimination performance decrements were seen under LFS instead of HFS stimulation seems to be somewhat unexpected at first glance. Motor benefits including the improvement of gait and postural deficits as well as faster response times are often seen under lower ‘therapeutic’ frequencies around 20–35 Hz [19, 22, 49] (but see [50]). In the parkinsonian monkey LSF of the PPN was shown to reduce akinesia [12]. Consistently, in the normal monkey high frequency stimulation (above 45Hz) caused akinesia, whereas low frequency stimulation (10–30 Hz) produced positive motor symptoms [10]. Cognitive improvements, if observed at all, were associated with LFS rather than HFS [51]. Explanations of the motor and cognitive improvement with LFS of the PPN are limited by our scant knowledge about the pathophysiological state of the PPN in PD [52, 53]. One possible explanation refers to overactive inhibitory projections from the globus pallidus pars interna (GPi) in the parkinsonian state [13, 52]. Those projections are assumed to cause over-inhibition of the PPN, with LFS relieving this tonic inhibition. HFS in contrast would accentuate the inhibition presumably because stimulation at 60Hz inactivates neurons by a depolarization block [3]. Following this model of PPN dysfunction in PD, it is PPN activation instead of inactivation that deteriorates discrimination thresholds. Of course, based on the present data we can only speculate about the type of modulations and pathways responsible for the observed response decrement under LFS. One possibility is that the performance decrement is mediated mainly by GABAergig projections of the PPN. GABAergig regions have been shown to segregate from cholinergic regions in the rodent [54]. Moreover, a division into a predominantly inhibitory (GABAergic) rostral portion and an excitatory (cholinergic/glutamatergic) caudal portion has been proposed to be a general organization principle of the PPN [55]. Stronger GABAergic inhibition of the SC due to stimulation in more rostral portions of the PPN could give rise to reduced visual discrimination performance. This would also be consistent with the better performance under LFS in patient P7, in which stimulation contacts were overall more caudal than in other patients showing performance decrements under LFS. However, patient P4, in which contacts were also more caudal, showed improved performance under LFS–a pattern that doesn’t align with this interpretation.

Alternatively, the performance decrement in the majority of our patients could arise from activated cholinergic projections of the PPN. Direct cholinergic projections to sensory gating structures like SC or LGN would then be unlikely to be responsible. Instead, the effect could be brought about via indirect modulatory pathways where PPN stimulation causes a downstream attenuation of the modulatory drive in attention gating structures. For example, the TRN, a thalamic structure receiving strong cholinergic projections from the PPN [30], is known to display high background activity thereby providing inhibitory modulation of the LGN [32]. Increasing the tonic excitation of the TRN could reinforce its inhibitory impact on processing in the LGN and consequently deteriorate discrimination performance.

As already mentioned, the present data cannot decide among the different ways of PPN’s potential influence on visual attention and sensory selection. A much better understanding of the physiological effects at the site of stimulation is required, and the pathways of PPN action need to be detailed in humans (see caveats below). Nonetheless, the present observations suggest that DBS of the PPN has an impact on visual selection processes at a rather elementary perceptual level, and that this influence depends on stimulation frequency.

### Caveats

While the present data provide some evidence for a specific influence of DBS on visual perceptual performance, they are at best preliminary and in demand of replication and further qualification. First of all, while the reported effects could be statistically validated, the number of subjects is fairly low. Confirming experimental evidence in a larger population of patients would surely be desirable. Unfortunately, it turned out that in the small group of reported PD patients DBS of the PPN was not associated with a significant improvement of gait and postural disturbances. Further surgical interventions in a larger patient group were therefore not justified.

Aside from our limited knowledge about the exact way DBS influences neural activity in the PPN, there are other limitations. One is the attainable specificity and precision of stimulating the PPN. The PPN is a small midbrain region in comparison to the spatial extension of the stimulation effect produced by the macroelectrode. It is therefore not unlikely that DBS in the PPN region affects neighbouring structures to some extent. In humans the PPN overlaps with the posterior part of the SN [56], so that it is presumably impossible to constrain stimulation to the PPN without also altering the SN [3]. Hence, the observed effects on discrimination performance may at least to some degree stem from a modulation of activity in the SN. The SN is a structure projecting to frontal cortex (FEF) involved in the control of visual attention [41–43]. Furthermore, at its caudal extreme the PPN is bordered by the locus coeruleus (LC)—a noradrenergic RAS nucleus with widespread efferent cortical and subcortical connectivity including projections to thalamic nuclei. Importantly the LC has been proposed to contribute to the selection of sensory information by modulating cortical and subcortical attention circuits [57]. Spill-over from PPN stimulation into the LC may therefore have effects on attention and arousal and influence the discrimination performance assessed in the present experiment. Finally as outlined in [3] 60 μs pulses as used in the present stimulation protocol may rather activate passaging fibres than neurons in PPN which would make an interpretation of the origin of the modulation effect problematic.
